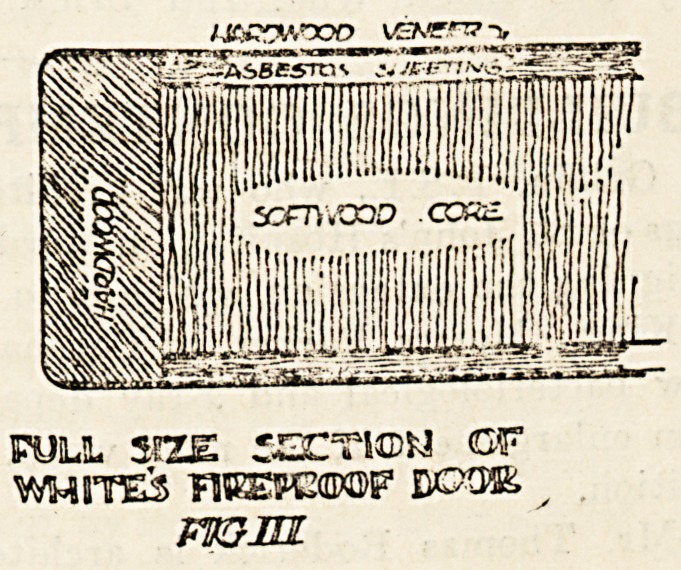# Hospital Doors

**Published:** 1911-08-19

**Authors:** 


### Hospital Architecture and Construction.

[Communications on this subject should be marked "Architecture" in the left-hand top corner of the envelope.]

### HOSPITAL DOORS.

In designing the fittings of hospitals an important item is the detail of doors and architraves. The detail which applies to house design is out of place in a hospital building.

The height of the average storey from floor to ceiling is about 12 or 13 feet, so that it will generally be possible to have a fanlight over the entrance door if found desirable. The necessary height of the doors is 7 feet 6 inches to 8 feet; the width varies from 2 feet 9 inches to about 5 feet 4 inches in the clear for a pair of doors such as at the entrance to the ward. Doors which swing both ways should always have a piece of glazing, however small, to enable persons proceeding in one direction to see those coming in the opposite direction, and thus to avoid awkward mishaps.

The material of which the doors and frame are made depends on the size and importance of the institution; in some cases deal or whitewood doors will be substituted for the more expensive hardwood, or possibly hardwood doors will be fixed in enamelled deal frames with enamelled architraves. Whatever the material, the rule as to simplicity of detail will apply.

The following sketch (fig. 1) gives a suggestion for a door which could be done either in deal or hardwood; the panel is glazed with fire-resisting glass set in copper, and a glance at the enlarged detail (fig. 2) shows there is no more moulding than is necessary. The thickness is $1\frac{3}{4}$ inch finished; this is the least allowable for an up-to-date fire resisting hardwood door.

The old-fashioned hinge and rising butts have now given place to the floor-spring, which can be adjusted so that the door closes slowly or quickly, depending on its purpose. The dimension from the top of the steelwork to the floor level is so small if a patent composite flooring is used that the greatest difficulty is experienced in finding a good working floor-spring, and a minimum thickness of about $2\frac{1}{4}$ inches should be provided. Nettlefolds have a good spring that works comfortably to this dimension.

Recently there has appeared on the market a

ELEVATION — SECTION

ENLARGED DETAIL

FULL SIZE SECTION OF WHITE'S FIREPROOF DOOR FIG III

composite door of which a detail is given in the accompanying illustration (fig. 3). It is made by John P. White, of Pyghtle Works, Bedford, and will no doubt meet with a favourable reception; a sample has a deal core 1⅜ inch thick, to which asbestos slabs are nailed and glued; the slabs are then levelled off and toothed ready to receive a hardwood veneer, which is glued and set under pressure. The advantage claimed for this door is its lightness compared with a solid panelled hardwood door. Its cost is 3s. 6d. per foot super, finished in oak, teak or mahogany.

## Figures and Tables

**Fig I f1:**
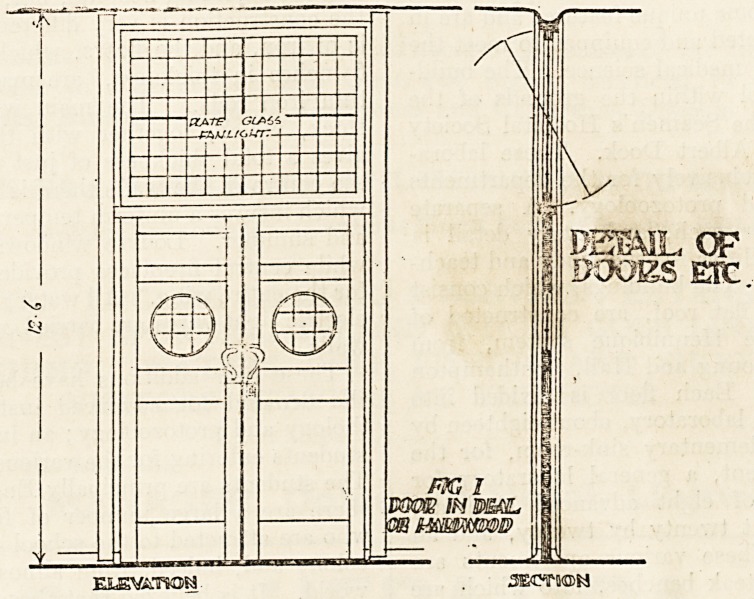


**Fig II f2:**
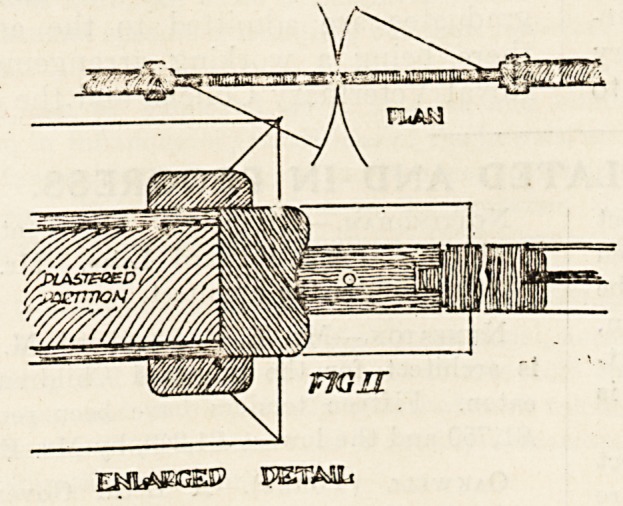


**Fig III f3:**